# Input-driven circuit reconfiguration in critical recurrent neural networks

**DOI:** 10.1073/pnas.2418818122

**Published:** 2025-03-07

**Authors:** Marcelo O. Magnasco

**Affiliations:** ^a^Laboratory of Integrative Neuroscience, Rockefeller University, New York, NY 10065

**Keywords:** recurrent neural networks, convolutional networks, unitary evolution, critical dynamics, circuit reconfiguration

## Abstract

Changing a circuit dynamically, without actually changing the hardware itself, is called reconfiguration, and is of great importance due to its manifold technological applications. Circuit reconfiguration appears to be a feature of the cerebral cortex, so understanding the dynamical principles underlying self-reconfiguration may prove of import to elucidate brain function. We present a very simple example of dynamical reconfiguration: a family of networks whose signal pathways can be switched on the fly, only through use of their inputs, with no changes to their synaptic weights. These are single-layer convolutional recurrent network with local unitary synaptic weights and a smooth sigmoidal activation function. We generate traveling waves using the high spatiotemporal frequencies of the input, and we use the low spatiotemporal frequencies of the input to landscape the ongoing activity, channeling said traveling waves through an input-specified spatial pattern. This mechanism uses inherent properties of marginally stable, dynamically critical systems, which are a direct consequence of their unitary convolution kernels: every network in the family can do this. We show these networks solve the classical connectedness detection problem, by allowing signal propagation only along the regions to be evaluated for connectedness, and forbidding it elsewhere.

## Significance

The brain has numerous mechanisms to modify its own circuitry. But physical alterations take time, and we have long known that interactions between neurons can change in fractions of a second during a task. Such dynamic reconfiguration, not based on physical changes, must instead be based on the dynamical state of the network. I present a minimal artificial recurrent neural network model, whose weights are unitary (critical). Inputs to the network can spatially pattern regions that are either supportive of, or refractory to, the propagation of neural traveling waves. The inputs to the network can therefore literally “draw” circuits in its pattern of ongoing activity, then erase them, then draw new ones, without ever changing the underlying “hardware.”

The brain displays an extraordinary amount of both early and life-long plasticity, achieved by modifying its own physical structure on vastly different timescales; for example, through synaptic plasticity (1), synaptic remodeling (2) and early and adult neurogenesis (3, 4). We have long known (5, 6) that some brain areas exhibit the ability to rapidly switch between several different functions depending on behavioral context, faster than would appear compatible with a physical change in the underlying neural substrate. It is now appreciated that the brain can dynamically reconfigure circuits without an underlying physical change, a wide phenomenon encompassing sensory areas (7, 8), motor areas (9, 10), and cognitive and decision-making areas (11, 12). One potential mechanism that has been strongly implicated in such rapid reconfiguration is ongoing brain activity (7, 13–18), for example ongoing traveling waves (19–22). Hence, as advocated in ref. 23, we aim to understand the dynamical principles through which a complex network can reconfigure itself through the management and curation of its own dynamical state.

I shall show below that recurrent networks in a critical state can be reconfigured rapidly and efficiently by suitable choice of inputs; and that the dynamical mechanism is that in such systems the network state has an inherently singular dependence on the input, and said state can permit or inhibit propagation of traveling waves in complex spatial patterns determined by the inputs. Conceptually, the inputs to the network, just like the inputs to any brain area, contain both ascending external sensory information as well as control signals from other brain areas (24); in other words, inputs naturally contain both data and programming. Information flows through critical recurrent networks along pathways which can be dynamically changed, with no changes to the synaptic weights, and without any special circuitry required to perform gating of interactions (11, 25).

I shall construct a set of examples, the simplest I could manage, where the input contains both control and signaling components in separate spatiotemporal bands. The signaling component (higher temporal frequencies) will consist of point-wise signal sources generating traveling waves of small amplitude; little ripples on top of the “ongoing activity.” The control component (low spatial and temporal frequencies) will literally be an image of walls and channels, and will drive the network into a state not unlike the copper layer in a printed circuit board: wherever the network ongoing activity is zero, the traveling waves move unhindered; wherever the ongoing activity is high, the traveling waves will be damped. Thus the waves will propagate into every place that is allowed by the input, implementing de facto a floodfill (“paint bucket”) algorithm, the classical algorithm to communicate information from one point to all connected points. This dovetails with a classical problem in neuroscience: how do the visual cortices detect connectedness of regions in visual stimuli (26, 27), a problem which is not solvable by feedforward architectures of fixed depth. It is argued cogently in ref. 28 that this is in fact a touchstone for the central role of recurrence in the nervous system.

It has been recently pointed out that RNNs trained in various memory-requiring tasks evolve traveling waves as transient memory storage (29) and as a means to communicate between network nodes (30). The hallmark of such traveling waves is a set of eigenvalues with unit absolute value. Our model shows that the ability to control such traveling waves through the dynamical state of the network, and thus through its inputs, is inherent to RNNs at their dynamical critical point (i.e., unitary dynamics). This ability does not require training, nor can it be gained or lost through training.

## 1. The Network

**Fig. 1.**
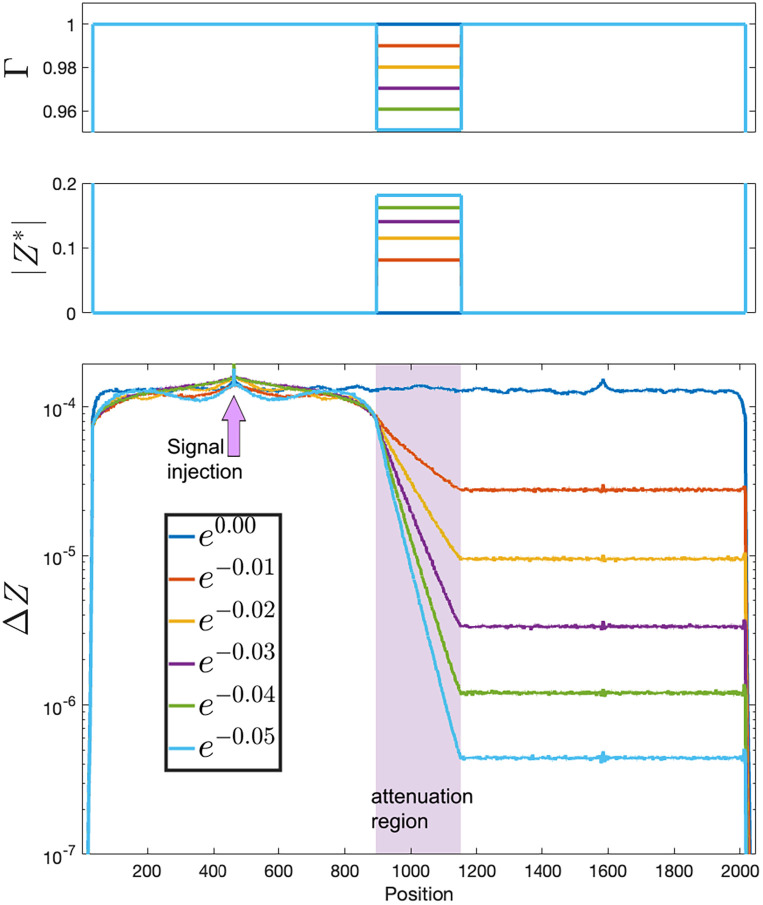
Wave propagation and attenuation in a 1D network (N = 2,048) with a nonuniform, temporally constant input $I^{*}$ giving rise to a spatially patterned attenuation array Γ. *Top* panel: Γ as a function of space; it has a value of 1 (nonattenuating) except for: First, the left and right flanks are strongly absorbent to prevent waves from wrapping around ($\Gamma_{\mathrm{walls}}=0.5$, offscale), and second, a range of positions at the center, called band, which was given six values of $\Gamma_{\mathrm{band}}=\;\exp\left(-0.01L\right)$, for L=0:5, dividing the spatial range into two boxes separated by an attenuation area. The signal (white noise) is injected at the center of the left box. Second panel: $I^{*}$ (real part, red; imaginary part, blue), computed from Γ through Eq. **9**. *Bottom* panel: |ΔZ| as a function of space, averaged, when a weak white noise signal is injected at the center of the left “box.” As the signal propagates outward from the injection site it traverses the $\Gamma_{\mathrm{band}}<1$ area at the center, where it attenuates exponentially, but reaches a new steady state on the right “box.” Please notice that the control over the attenuation is smoothly graded.

**Fig. 2.**
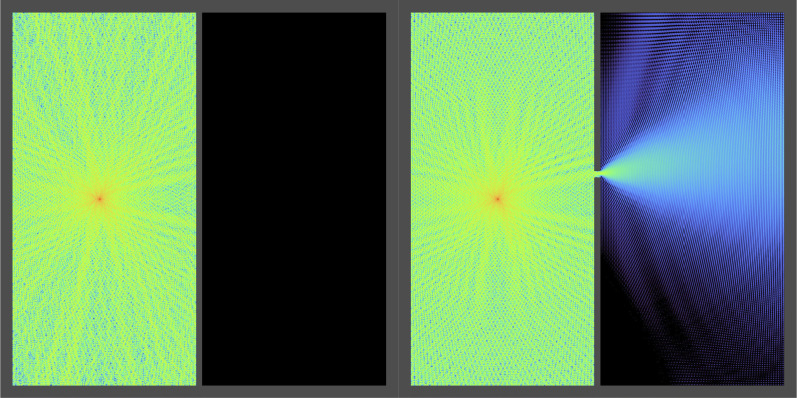
A 2D version of Fig. 1. The constant nonuniform $I^{*}$ input is used to pattern walls (here in gray) forming two boxes side-by-side. The gray areas attenuate traveling waves strongly and cannot be traversed. In addition to the input $I^{*}$ patterning the boxes, an additional oscillatory input is applied at the center of the left box. *Left*, the middle wall separating the two boxes is intact; the signal injected at the left box stays in the left box. *Right*, a small aperture is broken in the middle wall; the signal leaks into the right box. See the full movies in Movies S1–S3. This demonstrates the walls act as a geometric IF statement: something is allowed to happen, or not, depending on an input.

**Fig. 3.**
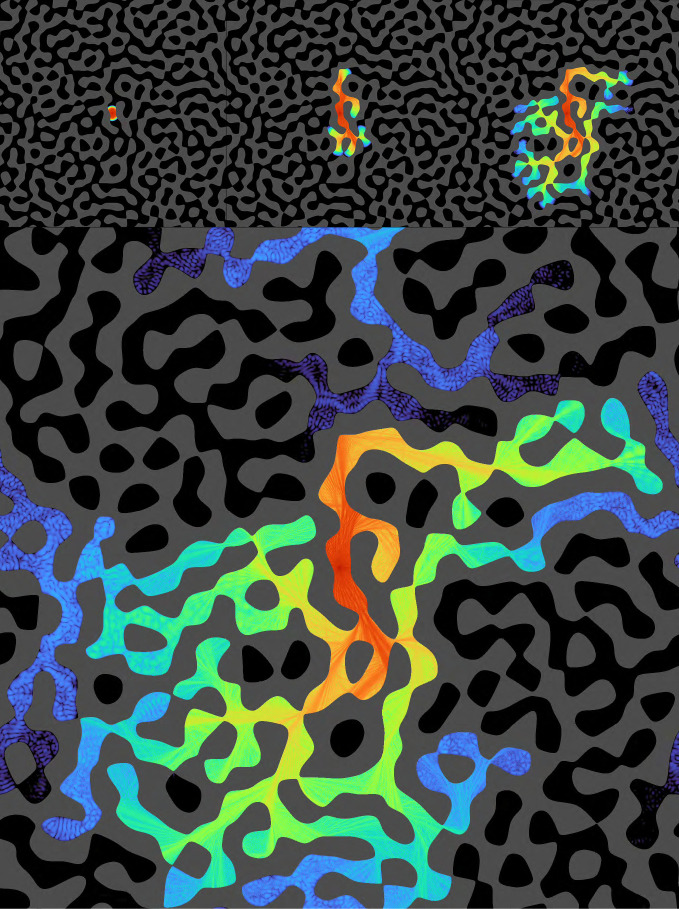
Four snapshots depicting stages in the outward propagation of an injected signal within an input-generated labyrinth, filling only the connected area and avoiding nonconnected areas; *Top Left*, briefly after injection; *Top Center*, *Top Right*, and *Bottom Panel*, subsequent evolution. A labyrinthine figure was created using band-passed noise to pattern a domain go, where propagation is allowed ($\Gamma_{\mathrm{go}}=1$, in black) and its complement nogo is strongly disallowed ($\Gamma_{\mathrm{nogo}}=0.01$, gray). An oscillating signal at the frequency of an eigenvalue was then injected at a single pixel at the center of the figure; the network was evolved and the panels show log(|Z|) in color code. Z is a 2,048 × 2,048 long complex array; U is the exponential of a numerical Laplacian kernel (times i). Please notice that the oscillatory signal did not fill noncontiguous areas, implementing the classic floodfill algorithm (a.k.a. *paint bucket*). Please find several full movies in Movies S4–S6.

I will use single-layer convolutional recurrent neural networks with unitary coupling kernels [cuRNNs (31)] and a smooth sigmoidal activation function. The state is given by a complex-valued neural layer Z; in this Paper, Z will be a 1D array of 2,048 complex numbers in Fig. 1, or a 2,048 × 2,048 2D array of complex numbers with periodic boundary conditions for Figs. 2, 3, and 5. We usually omit the spatial indices.

The state’s evolution in time is denoted as $Z_{n}$ where n is (integer) time, through the recursion

[1] $$Z_{n+1}=\phi \left(U⊗Z_{n}+I_{n}\right),$$

where ⊗ is the convolution operation natural to Z; U is a (unitary) convolution kernel (as defined below); $I_{n}$ is a sequence of external inputs of the same shape as Z; and ϕ is a smooth, scalar “activation function” operating element-wise on Z. A natural choice of ϕ for this construction is

[2] $$\phi \left(z\right)=z/\sqrt{1+{\left|z\right|}^{2}},$$

a complex-valued phase-preserving sigmoid (32).

We choose U to be a unitary kernel, so its eigenvalues lie on the unit circle; please see *Materials and Methods* for a discussion of the convolution in terms of linear algebra. The basic dynamical analysis of these networks is outlined in refs. 31 and 32; the family was derived as a Poincaré map from critical ODEs in ref. 33. We generate the unitary convolution kernel U by convolutional exponentiation of an anti-Hermitic kernel A (31):

[3] $$U=e_{⊗}^{A}\equiv F^{-1}\left\{\exp\left(F\left\{A\right\}\right)\right\},$$

where the exponential is element-wise, F and $F^{-1}$ the direct and inverse Fourier transforms in the dimension D appropriate to Z and A, and $A^{†}=-A$, i.e., anti-Hermitic.

We remind the reader that the eigenvalues of a convolution with a kernel U are the individual elements of the Fourier transform of U, and the corresponding eigenvectors are the Fourier modes associated to those elements; please see *Materials and Methods* below for a discussion. The Fourier transform of the kernel A giving rise to U is called the dispersion relation; since it is purely imaginary, it associates a nondecaying frequency to each wavenumber. In Physics, dispersion relations are expected to be continuous and reflect the underlying PDE. Here the dispersion relation may be arbitrary and discontinuous.

## 2. Signal Propagation

The propagation of perturbations through this system can be analyzed in terms of linear stability of dynamical systems (34, 35). The derivative of the state $Z_{n}$ at time n with respect to the initial state $Z_{0}$ at time 0 telescopes through the chain rule into a product of derivatives (34, 35), each one of which contains two terms: the constant unitary matrix representing the action of the convolution with U (denoted by [U⊗] in ref. 31 and in the M&M below), times the matrix of derivatives of ϕ evaluated at its argument at the appropriate time-step; since ϕ is element-wise, this is a diagonal matrix:

[4] $$\frac{\partial Z_{n}}{\partial Z_{0}}\approx \prod_{i=0}^{n-1}\Gamma_{i}\cdot \left[U⊗\right]\;\mathrm{where}\;\Gamma_{i}≐\mathrm{diag}\left[\phi^{\prime }\left(U⊗Z_{i}+I_{i}\right)\right].$$

If ϕ is a sigmoidal function, and satisfies ϕ(0)=0, $\phi^{\prime }\left(0\right)=1$ and $0<\phi^{\prime }\left(x\right)<1\;\;\forall x\neq 0$, then for I=0: a) the state Z≡0 is globally attracting (i.e., all activities in the layer decay to 0 regardless of initial conditions), and b) the system is “critical” in that the derivative above is a power of the constant unitary matrix and, thus, unitary itself; all eigenvalues have unit absolute value, and therefore the state is marginally stable (critical), at the onset of a large number of Hopf bifurcations. Perturbations away from Z≡0 decay toward zero slower than exponentially, just algebraically, but they do decay. In dynamical systems terms, this system has a spanning set of (weakly attracting) center manifolds (34).

Unitarity of the coupling matrix has many other implications beyond the dynamical ones we explore here. In particular unitary couplings have been employed in the ANN literature, e.g., refs. 36–40, as a means to overcome the vanishing gradient problem (41). The fact that the same matrix Γ·[U⊗] controls both asymptotic stability as well as backpropagation is a connection that will be explored elsewhere. Another connection is that traveling waves are directly associated to such unitary modes through circulant matrices (30).

For $\phi \left(z\right)=z/\sqrt{1+{\left|z\right|}^{2}}$, input forcing at an eigenfrequency of U yields the singular cubic-root compression characteristic of the forced Hopf bifurcation (32, 42, 43), where the response amplitude scales with the cubic root of the input amplitude at resonance.

Please find in *Materials and Methods* a discussion of the scalar case. We will now analyze a spatially extended system. We first review the case already considered in ref. 32: a spatially and temporally constant input generates a uniform steady state; an oscillatory perturbation injected into a single site generates waves that propagate outward from the injection site and attenuate as they spread. The constant component creates ongoing activity, causing the operating point of the dynamical system to shift to values of Z for which the derivative of ϕ is no longer 1. The oscillatory input then propagates against this background.

We write the input as $I_{n}=I^{*}+\alpha \lambda^{n}\delta_{0}$, where $I^{*}$ is a spatially and temporally constant input, $\lambda =e^{i\theta }$, and $\delta_{0}$ the image with a single 1 at element (0,0). Following standard dynamical-systems perturbation analysis, we will analyze the behavior around α→0, and so henceforth we assume $\alpha \ll \max|Z^{*}|$. For α=0 our system reaches a steady state given by

[5] $$Z^{*}=\phi \left(U⊗Z^{*}+I^{*}\right),$$

where it follows that $Z^{*}$ is spatially and temporally constant, and obeys the scalar relations in Fig. 8.

For α≠0 we write $Z_{n}=Z^{*}+\Delta Z_{n}$ into Eq. **1** to obtain

$$Z^{*}+\Delta Z_{n+1}=\phi \left(U⊗Z^{*}+U⊗\Delta Z_{n}+I^{*}+\alpha \lambda^{n}\delta_{0}\right)$$

and expanding the RHS to first order we obtain

[6] $$\begin{array}{cc} Z^{*}+\Delta Z_{n+1} & =\phi (U⊗Z^{*}+I^{*})+\cdots \\ & \;+\phi^{\prime }\left(U⊗Z^{*}+I^{*}\right)\left(U⊗\Delta Z_{n}+\alpha \lambda^{n}\delta_{0}\right) \end{array}$$

the first terms in both the right-hand side and left-hand side cancel, and calling Γ the value

[7] $$\Gamma ≐\phi^{\prime }\left(U⊗Z^{*}+I^{*}\right)=\phi^{\prime }\left(\phi^{-1}\left(Z^{*}\right)\right)$$

we reach a linear propagation equation for the perturbation generated by the oscillatory input:

[8] $$\Delta Z_{n+1}=\Gamma \cdot \left\{U⊗\Delta Z_{n}+\alpha \lambda^{n}\delta_{0}\right\}$$

from where we see that the wave generated by the oscillatory input attenuates by a factor of Γ every time-step. This temporal attenuation becomes a spatial attenuation due to the finite speed of propagation of the wave (its group velocity): the slower the wave, the many more factors of Γ are incurred in traversing a given distance.

As shown in ref. 32, the asymptotic state for this equation can be computed in closed form. Asymptotically ΔZ will relax onto a periodic solution with the frequency λ of the forcing and some overall space-dependent amplitude $R^{*}$. Writing $\Delta Z_{n}=R^{*}\lambda^{n}$ and substituting in the equation above we get $R^{*}\lambda =\Gamma \left(U⊗R^{*}+\alpha \delta_{0}\right)$, an equation which is explicitly solvable in Fourier space

$$R^{*}=F^{-1}\left[\cdot \frac{\alpha F[\delta_{0}]}{\lambda /\Gamma -F[U]}\right],$$

where ·− denotes element-wise division. For Γ→1 as λ approaches an eigenvalue of U (these are, in fact, the elements of F[U] and are, as discussed arranged on the unit complex circle) the denominator has a pole and the corresponding eigenvector dominates $R^{*}$, but for Γ<1 the denominator is bounded away from 0 and the responses are spatially decaying exponentials around the injection point.

## 3. Spatial Patterning

The previous discussion suggests to use a spatially varying $I^{*}$ to geometrically pattern areas of the array where a signal is not allowed to enter and others where it is free to propagate. Given a temporally constant but spatially patterned input $I^{*}$, iteration of Eq. **1** will reach a spatial pattern of ongoing activity $Z^{*}$ given by the fixed point Eq. **5**. Perturbations around the fixed point evolve according to Eq. **8**, through the derivative of the activation function ϕ evaluated at its arguments $U⊗Z^{*}+I^{*}$; this derivative $\Gamma ≐\phi^{\prime }\left(U⊗Z^{*}+I^{*}\right)$ is now spatially patterned and will generate areas of unobstructed wave propagation wherever |Γ|=1, wave absorption whenever |Γ|<1, wave amplification and regeneration when |Γ|>1, and complex scattering effects when the phase of Γ varies spatially.

To engineer a given outcome we have to work our way backward through the equations: first, from the desired Γ, we compute the $Z^{*}$ that gives rise to that Γ; second, we compute the $I^{*}$ that gives rise to $Z^{*}$.

Start with the construction of the desired spatially varying attenuation Γ: this is an array of the same size and shape as the layer Z, and in the spatial locations where |Γ|=1, waves propagate unhindered, and wherever |Γ|<1, waves attenuate. Then we invert Eq. **7** to get the arguments of $\phi^{\prime }$ as a function of Γ. For our choice of ϕ, we have $\phi^{\prime }\left(z\right)={\left(1+z^{2}\right)}^{-3/2}$ so $z=\sqrt{{\left(\phi^{\prime }\right)}^{-2/3}-1}$. (Please note that this last expression depends on the functional form of ϕ). From there,

$$U⊗Z^{*}+I^{*}=\sqrt{\Gamma^{-\frac{2}{3}}-1}$$

one equation with two unknowns, $Z^{*}$ and $I^{*}$. To solve it we refer to Eq. **5**, $\phi \left(U⊗Z^{*}+I^{*}\right)=Z^{*}$: if we apply ϕ to both sides, the left side becomes $Z^{*}$ and the value of $I^{*}$ disappears giving us directly the value of $Z^{*}$ as a function of Γ:

$$Z^{*}=\phi \left(\sqrt{\Gamma^{-2/3}-1}\right).$$

For the second step, we solve Eq. **5** for the input $I^{*}$: $I^{*}=\phi^{-1}\left(Z^{*}\right)-U⊗Z^{*}$, and substituting the former expression into the latter we obtain

[9] $$I^{*}=\sqrt{\Gamma^{-2/3}-1}-U⊗\left[\phi \left(\sqrt{\Gamma^{-2/3}-1}\right)\right].$$

Eq. **9** is our central result. Using this equation we can obtain the values of the input that is needed to cause any desired pattern of spatial attenuation.

In Fig. 1, we show how spatially patterned attenuation works in a 1D array. A set of neurons (call it band) at the center of the array will have values $\Gamma_{\mathrm{band}}<1$, implemented by using Eq. **9** to get a pattern of ongoing activity (where $\Gamma_{\mathrm{set}}=\alpha$ is shorthand for $\Gamma_{i}=\alpha \;\forall i\in set$. the values of the array Γ, for every neuron in the set set, equal the scalar value α). Signal will be injected at a single point on the left of this area, and will propagate outward; as it goes through the central area band it will attenuate exponentially in space, and will reach a steady state on the right.

In Fig. 2, we generalize the idea of Fig. 1 to 2D, by now crafting two boxes separated by a wall, but instead of having a band of continuous attenuation, we make a small hole in the wall. We use a (complex) layer Z of size 2,048 × 2,048, and $U=e_{⊗}^{i\Delta }$ with Δ a finite-difference 3 × 3 Laplacian kernel. An array Γ was created defining two boxes stacked side-by-side; the walls of these boxes are strongly attenuating ($\Gamma_{\mathrm{walls}}=0.1$) and cannot be traversed by signals, while the inside of the boxes has $\Gamma_{\mathrm{inside}}=1$ allowing waves to propagate freely. From Γ we computed $I^{*}$ using Eq. **9**. An oscillatory signal was then added to $I^{*}$, applied at a single pixel at the center of the left box. When the wall between the boxes is intact, the signal at the left cannot reach the right box. If, on the other hand, a small hole is made in the separating wall, then the signal reaches the right. Therefore this shows how the input signal $I^{*}$ determines whether or not other signals are able to propagate; in effect, this is a geometric IF statement that allows something to happen, or not happen, depending on the input values.

In Fig. 3, we used the same complex-valued 2,048 × 2,048 layer Z and kernel U; created a labyrinthine pattern (using thresholded band-pass noise) to create “go” and a “no-go” regions, and mapped it to Γ by $\Gamma_{\mathrm{go}}=1$ and $\Gamma_{\mathrm{nogo}}=0.1$, and computed the $I^{*}$ and $Z^{*}$ that generate Γ. Again an oscillatory signal was applied, this time at the center of Z. The signal propagates only along the allowed channels, filling the go area which is connected to the center pixel, but not invading noncontiguous regions. This implements floodfill, a basic algorithm for detection of connectedness that, as discussed above, has been shown not to be computable using feed-forward networks of finite depth (26–28).

**Fig. 4.**
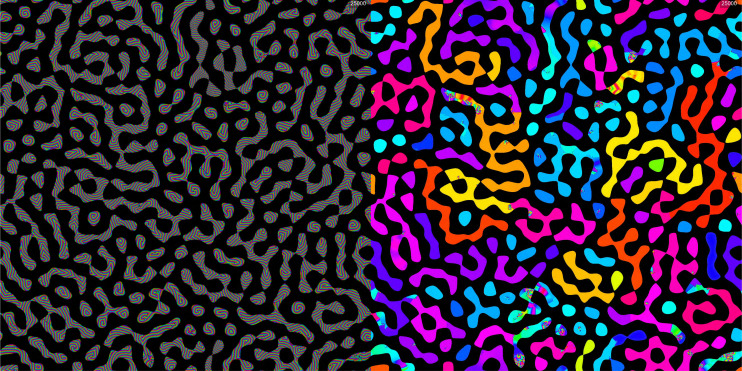
Binding connected domains by synchrony of oscillations. A labyrinthine patterns is created as described and used to generate an array Γ which takes two values: 1.2, generating spontaneous oscillations, and 0.2, strongly damping. $\Delta Z_{0}$ is initialized to contain very small random Gaussian numbers; 25,000 iterations of the map are taken. *Left*, the complex-values of $\Delta Z_{25,000}$ are shown with the color indicating complex phase. *Right*, a map of the frequency of oscillation of individual neurons shows that at time n=25,000 most domains are well on their way to being fully synchronized, with several domains still showing synchronization boundaries. (The frequency is evaluated as the phase difference between $Z_{25,000}$ and $Z_{24,800}$) The full movies are available in Movie S7.

The traveling waves used as tracing signals to map connectivity may not need to be external inputs, but can also be generated endogenously. In Fig. 4, we have taken again the same network, generated a boolean image with a labyrinthine pattern, converted said image into a Γ and from there into an $I^{*}$ as already described. But in this case, instead of using a purely conducting value of $\Gamma_{\mathrm{go}}=1$ we used a supracritical amplifying value of $\Gamma_{\mathrm{go}}=1.2$. As a result, the system spontaneously oscillates and generates endogenous waves. However, the waves cannot traverse the γ=0.1 areas. So the endogenous waves are confined to bounce around within each connected area, and after transients all the neurons in the area synchronize to the same frequency; for the particular kernel that we used this results in a classic wave pattern. Because the kernel has a number of different frequencies available, the selected frequency is influenced by the shape of the connected domain and also somewhat by chance during the spontaneous oscillation growth. The result, as shown in Fig. 4 is that each domain oscillates at a different frequency, pretty much instantiating the ideas of perceptual binding using synchrony.

**Fig. 5.**
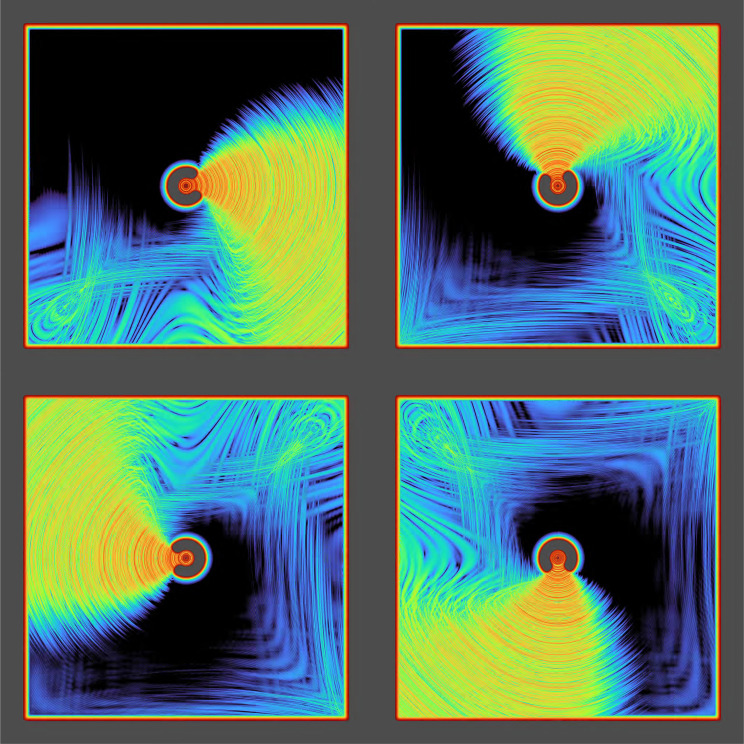
The “Lighthouse.” This figure demonstrates directional wave emission changing in time. A slowly varying $I^{*}$, in the shape of an annulus with 1/4 turn deleted, slowly rotates; a signal source is placed at its center, and the generated waves can only escape through the gap, and continue to propagate in the same direction. The outer walls have carefully tapered values of Γ to slowly absorb the waves preventing their reflection, and the central annulus is itself carefully smoothed so that waves are not emitted from any sharp turning borders. Please find the full movie in Movie S8.

As an example of a moving boundary condition, in Fig. 5, we used as an input, a slowly rotating image of 3/4 of an annulus (i.e., an annulus with one quarter missing) as our impenetrable wall, and placed a signal source at its center. As a result, the signal escapes the annulus through the gap; signals continue propagating in the direction they were emitted from. As the annulus slowly rotates, it emits signals all around it, forming a little lighthouse. This demonstrates input-dependent directional selection of a signal, or dynamic routing.

## 4. Generalizations

We now proceed to examine which assumptions we can relax and how general this phenomenology is. The necessary condition for our results to follow is that the system is at a dynamically critical point with weakly contracting center manifolds. Let us examine what this condition means and what is required to meet it.

In the absence of input (so $I_{n}\equiv 0\;\forall n$), Eqs. **1**–**3** together define a dynamical system with a marginally stable, globally attracting fixed point. The state Z=0, where all neurons of the layer are quiescent, is a fixed point of the equations: 0=ϕ(U⊗0). The eigenvalues of the derivative matrix (Hessian) $\partial Z_{1}/\partial Z_{0}$ (Eq. **4**) all have absolute value equal to 1, so the linear stability (34) of the system is marginal and undecided to linear order. But ϕ is contracting and all nonzero distances between points are made smaller by its application: |ϕ(a)−ϕ(b)|<|a−b| if a≠b. Therefore Euclidean distances between two arrays $Z^{a}$ and $Z^{b}$ are made smaller: $|\phi \left(U⊗Z^{a}\right)-\phi \left(U⊗Z^{b}\right)\left|<\right|Z^{a}-Z^{b}|$, and the Banach fixed point theorem states there is a unique stable and globally attracting fixed point. So this is a system poised at the critical point of a large number of Hopf bifurcations. In jargon, this system has a high-dimensional stable center manifold.

The Ruelle-Takens-Newhouse theorem (44, 45) states that a generic dynamical system with more than three simultaneous Hopf bifurcations is not resilient to perturbations of its defining equations; and it is in fact more fragile the higher the number of bifurcations. As applied to our equations then it would seem our system would be massively fragile to perturbations in the defining dynamics: Figs. 2, 3, and 5 all contain 4-million-dimensional tori. But recurrent neural networks in general and Eq. **1** in particular are members of a family of dynamical systems which includes coupled map lattices (in discrete time) and reaction–diffusion equations (in continuous time). In these systems the coupling structure is linear and all nonlinear interactions occur locally, as a consequence of locality rules imposed by the underlying physics or physiology. If we only consider perturbations to the dynamics that have to obey locality, some dynamics that are structurally unstable in a generic ODE or map may be possible in an RNN.

Our ϕ satisfies ϕ(0)=0 (the fixed point is at 0), $\phi^{\prime }\left(0\right)=1$ (slope 1 at the fixed point) and $\phi^{\prime }\left(z\right)<1\forall z\neq 1$ (contracting everywhere other than the fixed point). In general, given a smooth sigmoidal activation function, we can change coordinate system to center the fixed point and then factor out the slope of the activation function and absorb it into the synaptic matrix, to make the slope at the fixed point 1. To be clear, “unitary dynamics” really has no meaning unless $\phi^{\prime }\left(0\right)=1$ as Eq. **4** multiplies them together. Not every sigmoidal activation function will be guaranteed to be globally contracting after this translation and rescaling because the original fixed point is not guaranteed to be at the point of maximal slope. (Please remember that the expression in Eq. **9** explicitly incorporates the form of $\phi^{\prime -1}$ and will have to be rederived for a different ϕ).

Movies S4 and S5 show the difference between injecting a resonant oscillation vs. white noise. There is no fundamental qualitative difference.

Our U needs to be the convolutional exponential of an anti-Hermitian kernel A with compact support (i.e., local); if the radius of the kernel A exceeds the size of the gaps between connected regions, then the activation can “tunnel” across. Figs. 3–5 have been produced using the same kernel, $U=e^{i\Delta }$, to emphasize the fact that the connections did not change, only the input did; but any compact kernel A will work. Movie S6 shows the floodfill dynamics using a random anti-Hermitian kernel within a disk of radius 3 (i.e., a 7 × 7 kernel with rounded corners). This random kernel contains both symmetric (purely imaginary) and antisymmetric (real) components. It is important to note that Eq. **9** depends on the kernel, so changing the kernel means changing the input required to achieve a desired Γ; but given this, the results are independent of the kernel. Therefore these features can neither be acquired nor lost by training so long as unitarity is maintained.

The set of networks satisfying these assumptions is quite large within the space of all cuRNNs so the ability to control wave propagation is quite generic in this space. The biggest assumption left to relax is translational invariance, i.e., the convolutional nature of the network. One of the main reasons for this assumption is practical: this paper has illustrated the evolution of systems with 4 million (complex valued) neurons for millions of timesteps, a system that would be very expensive to simulate for a general RNN. Convolutional systems are fast.

On the other hand, a 1D RNN with N = 2,048 is easy enough to simulate. However, 1D systems with local random connections suffer from a phenomenon called “Anderson localization,” whereby the support of some eigenvectors collapses into local lumps and does not span the system size; even if the system is unitary it does not support global traveling waves as they are confined within the support of the eigenvectors. To avoid this problem we generate an anti-Hermitian 1D synaptic matrix A containing a sum of two terms: a translationally invariant 1D wave kernel, and a second term consisting of set of six off-main diagonals containing random, i.i.d., Gaussians (at −3,−2,−1,+1,+2,+3 from the center diagonal). If the weight of the second (random, nonconvolutional) term is sufficiently smaller than the first, the system has some localized and some system-spanning eigenvectors.

The evolution is given by

$$Z_{n+1}=\phi \left(UZ+I_{n}\right),$$

where now U is the matrix exponential of A and UZ is matrix multiplication. The analog of Eq. **9** then becomes

[10] $$\begin{array}{cc} Z^{*} & =\phi \left(\sqrt{\Gamma^{-2/3}-1}\right),\\ I^{*} & =\phi^{-1}\left(Z^{*}\right)-UZ^{*}. \end{array}$$

**Fig. 6.**
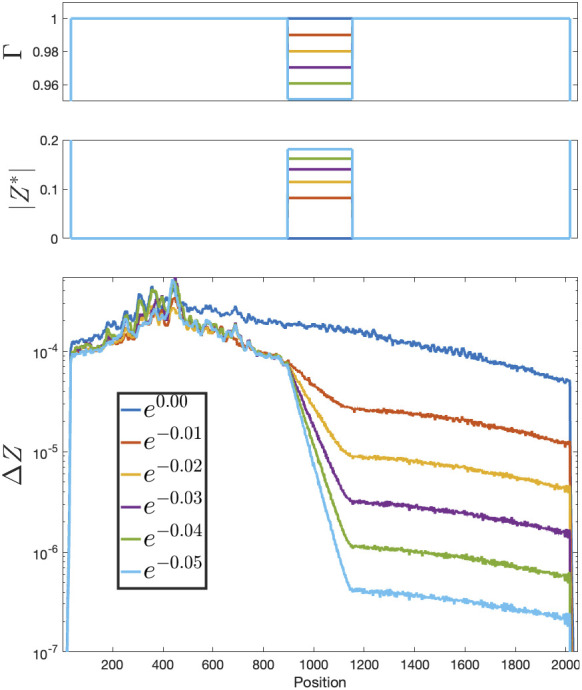
The phenomenology of a 1D nonconvolutional uRNN with local connections is not different from that of a cuRNN. Here we have an RNN with N = 2,048 whose connection matrix is generated from an anti-Hermitian matrix as described in the text. Exactly the same setup as in Fig. 1. *Top* panel, Γ is 1 except for absorbing left and right wall, plus a band with Γ<1 at the center, which divides the layer into two. Weak white noise signal is injected at the center of the left “box” and has to travel through the attenuating area to reach the right side. *Middle* panel, $I^{*}$ computed using Eq. **10**. *Lower* panel, waveform amplitude after 65,536 iterations (averaged over the last 1,024 iterates); *Inset* panel shows value of γ corresponding to each curve color. As the waves spread from the center they enter the attenuation regions and their amplitude drops exponentially with distance traveled within the region. Notice that even for Γ=1 the outgoing wave seems to attenuate slightly as it travels: energy is getting absorbed into Anderson-localized eigenvectors. The amplitude of the random component was chosen to be small enough to generate roughly 1/2 localized and 1/2 globally spanning eigenvectors.

We can observe in Fig. 6 the same phenomenology as in the convolutional case: a signal injected at the center of the left box propagates through regions with Γ=1 and attenuates exponentially upon penetrating regions with Γ<1. However a slight attenuation of the waveform is visible away from the injection point even when Γ=1 because energy is lost into localized eigenvectors. At higher amplitudes of the random component the propagation altogether collapses. Code in *SI Appendix*.

Therefore, with some degree of care and additional caveats, nonconvolutional uRNNs display the same ability to control wave propagation through ongoing activity.

## 5. Discussion

*Perhaps the most striking aspect of the findings reported here is the apparent ability of the cortex to dynamically modify the processing of visual information according to immediate behavioral requirements.*

Christ and Gilbert, in ref. 5 (2001)

A key point of experimental design enabled the observation reported in ref. 5: the macaque initiated trials by pulling a lever attached to the primate chair. Thus the experimental measurement spanned a transition in behavioral state. An experiment which is locked into one behavioral state may miss circuit reconfigurations as they occur during these transitions.

Our view of how the brain operates has evolved considerably in the years since, resulting in an increasing intellectual tension between the circuit view and the functional connectivity view (11, 25). For example, ref. 7 observes considerable changes in the extent of lateral spreading of activity in V1 as a function of the background contrast of the images presented; at low contrast activity spreads for a considerable distance on the cortical surface, while for high contrast the activity does not spread. The authors then postulate the existence of gating circuitry that will allow this change in interaction strength to happen, which has not yet been found. Meanwhile, as shown in ref. 46 using antisymmetric ODEs, a critical system can change such spatial scales dynamically without an explicit gating device.

In more recent years, the advent of high channel count electrophysiology has led to a new paradigm in how we conceptualize brain activity. Before, a prevalent notion was that brain activity was supposed to be high dimensional, because otherwise why would we have so many neurons? But modern high-dimensional electrophysiology observes that the simultaneous activity of many neurons together appears to lie on relatively low dimensional hyperplanes or neural manifolds (9, 11, 25, 47–50); the catch being that changing the task changes the manifold. For example, in motor control of arm reaching, the concerted activity of many neurons appears to be confined to a plane where the dynamics is rotational; however, different reach motions generate different planes (10). This leads to a view of the cortex as capable of generating a large number of different low-dimensional manifolds on demand: effectively, a machine of creating dynamical systems.

As a result, our understanding has evolved into a view where brain activity is far more labile and pliable than a naive connectivistic view would imply; and so the field has repeatedly asked: where are the gating circuits enabling this flexibility? The work presented here shows that there is no logical need to have “a gating circuit.” Such circuits may exist anyway, but perhaps one should not be alarmed if they are not evident.

## 6. Conclusions

To conclude, we have presented a minimal model of dynamic reconfiguration. The model is inherently geometrical, because it is laid out on an Euclidean lattice supporting convolutions, but illustrates concepts that can be used on any RNN: a) to use unitary synaptic matrices that support traveling waves, b) to exploit the inherent sensitivity of such a system to sculpt ongoing activity, c) and to use that activity to dynamically control the space through which the traveling waves can move. This mechanism can be viewed as a geometric form of an IF statement: depending on some data (the input), a computation is either carried out, or not; in this case a transfer of information via a traveling wave. The ways that such a geometrical construct could be exploited might be many. But as we have shown, this mechanism is not only to drive local activity to saturation to create a patch of refractory tissue; as Figs. 1 and 6 show, this is a continuously graded mechanism.

We make no representation that this model is optimal in any way other than being conceptually simple, nor we make a claim that it is directly applicable to computational neuroscience other than illustrating a fundamental dynamical mechanism. No effort was made to optimize or backpropagate any of the available degrees of freedom: the kernel, the activation function, or the patterns used as input. It is clear that a designed or optimized network could do materially better at the tasks presented. The virtue of this model is fundamentally one: Figs. 3–5 were run on the same, identical network: same kernel, same activation function. The only thing that changes between Figs. 3–5 is the input $I^{*}$ given to pattern the tasks, and the location of the signal sources. (Movie S7 uses a random 7 × 7 kernel, specifically to illustrate that the results are not sensitive to the choice of kernel). In fact, if separated from each other by adequate pauses to “reset” the ongoing activity and waves, all such inputs could be concatenated sequentially in one single simulation.

Artificial neural networks are well known to be difficult to “interpret,” as we lack analytical methods to parse their huge number of parameters into an explanation of what they are doing. Recurrent neural networks are particularly difficult in this regard. Our results show that the ability to control the propagation of traveling waves is inherent to RNNs: as traveling waves can only be supported by unitary eigenvalues, the critical dynamics of such eigenvalues immediately allows for them to be controlled in the manner described above. No special trick is required, no additional “gating” circuit is necessary. If traveling waves were rare, this Paper would be unneeded. But traveling waves have been persistently observed in brain activity, and recent computational results (29, 30) show that training RNNs to perform tasks requiring long working memories (such as sequence learning) causes them to spontaneously develop traveling waves to instantiate such memory and communicate between processing nodes in the RNN. So it may be that traveling waves are, in fact, a fundamental integrative process of neural dynamics; if so, this work presents a natural way to control them.

## Materials and Methods

### Expressing Convolutions as Linear Algebra Operations.

A standard form recurrent network is expressed as

[11] $$X_{n+1}=\phi \left(MX_{n}+I_{n}\right),$$

where the activities of the neurons are collected in a vector X, M is the synaptic weight matrix, MX is matrix multiplication, and n discrete time.

**Fig. 7.**
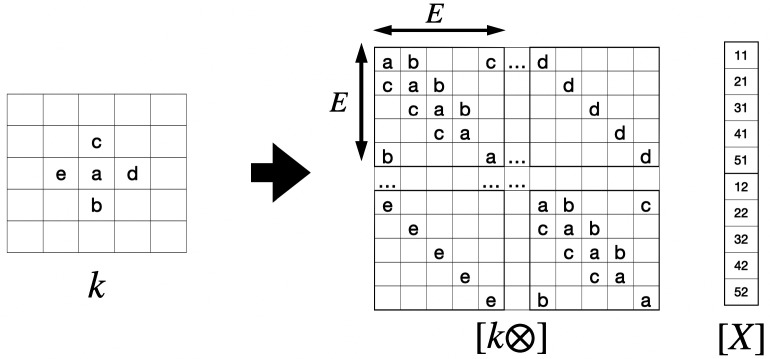
The structure of [k⊗] for a 2D convolution kernel k when the layer X has dimensions E×E, column concatenation basis. The array X is represented by a vector [X] of length $e^{2}$. Consider the convolution kernel k having only 5 nonzero coefficients abcde in the geometric pattern shown, with a at the center. (The classic Laplacian would be written b,c,d,e=1/4, a=−1). The [k⊗] matrix is now $E^{2}\times E^{2}$, and is composed of an E×E circulant arrangement of blocks; each block is a circulant matrix of dimension E×E. From ref. 32.

If all elements of M are nonzero we say this network is fully connected; but if M has some special structure then we get a specific subtype of RNN. For example, a system having two different layers R and S can be expressed by an X=(R,S), where (,) is concatenation, in which case the matrix M will have a 2 × 2 block structure with self-connections on the diagonal blocks and cross connection off-diagonal. Similarly, a system having a second-order-in=time dynamics $V_{i+2}=\phi \left(PV_{i+1}+QV_{i}\right)$ can be expressed by defining $W_{i}=V_{i+1}$ so then

[12] VWi+1=χ0QIPVWi

and χ is the identity function on the top block and ϕ on the bottom block.

For reasons of analysis we would like to express a convolutional network $Z_{n+1}=\phi \left(K⊗Z_{n}+I_{n}\right)$ in the same form, since clearly the only difference is that the weights in K are being recycled, so we should get a matrix M with some kind of special repetitive structure. This entails expressing Z as a vector, regardless of its shape. The equivalent of the matrix M is not just K, but rather K⊗, since it is the application of K through convolution that parallels matrix multiplication. Expressing Z as a vector is obvious when Z is a 1D array, but if Z is 2D or above, we need to reshape it, e.g., by concatenating rows or by concatenating columns. In general, we will choose a basis B for Z and call ${\left[Z\right]}_{B}$ the representation of Z in the basis B. Once we have chosen B we can then proceed to represent ${\left[K⊗\right]}_{B}$: this is the matrix representation we have been looking for. Eliding the B we get

$${\left[Z\right]}_{t+1}=\phi \left(\;\left[K⊗\right]{\left[Z\right]}_{t}+{\left[I\right]}_{t}\;\right)$$

which is now explicitly in the form of Eq. **11**. What kind of structure does [K⊗] have? In 1D, if the layer does not wrap around, it has constant diagonals and is known as a Toeplitz matrix; if the layer wraps around so there is an $M_{1n}$ element, it is a circulant matrix. In 2D with periodic boundaries it acquires a block structure of circulant matrices as shown in Fig. 7.

Once we understand the process K→[K⊗] we see that we can attribute to K any property of [K⊗] that is invariant under change of basis, such as eigenvalues or unitarity. Besides unrolling, the other basis we need to consider is B=F, the Fourier basis. All convolutions are diagonal in the Fourier basis, which leads to the diagonal of ${\left[K⊗\right]}_{F}$ being multiplied elementwise by ${\left[Z\right]}_{F}$; since said diagonal is the Fourier transform of K (in the dimension appropriate to K) we get ${\left[K⊗\right]}_{F}=\mathrm{diag}\left(F\left[K\right]\right)$, where we have abused notation by naming the Fourier transform and the Fourier basis with the same letter) also known as the convolution theorem.

### Linear Stability and the Cubic Root.

The steep cubic root nonlinearity is important for our argument, so we will review its origin here, in a scalar example where the layer has one single neuron:

$$z_{n+1}=\phi \left(z_{n}+I_{n}\right)=\frac{\left(z_{n}+I_{n}\right)}{\sqrt{1+\left(z_{n}^{2}+I_{n}\right)}},$$

where z is now one single complex number and so is $I_{n}$. For constant I, this recurrence converges to a fixed point $z_{n}\to z^{*}$ (we will follow henceforth the convention of using the superscript ^∗^ to denote a quantity at a fixed point) which is given by $z^{*}=\phi \left(z^{*}+I\right)$. For small values of I then $z^{*}={\left(2I\right)}^{1/3}$: the value of $z^{*}$ depends very sensitively and nonlinearly on I, as the cubic root amplifies small values enormously. This is because ϕ(z) is tangent to the diagonal line at 0; when adding I to the argument of ϕ causes the curve to translate left by I, and the fixed point, which is given by the intersection with the diagonal line, immediately darts up and right. This effect is illustrated in Movie S1. If we then consider the evolution of a small perturbation $\delta z_{n}$ around $z^{*}$, following standard stability analysis it will evolve (34, 35) through

$$\begin{array}{cc} z^{*}+\delta z_{n+1} & =\phi (z^{*}+I+\delta z_{n})\\ & \approx \phi \left(z^{*}+I\right)+\frac{d\phi }{\mathrm{dz}}\left(z^{*}+I\right)\;\delta z_{n}+O\left(\delta z^{2}\right). \end{array}$$

**Fig. 8.**
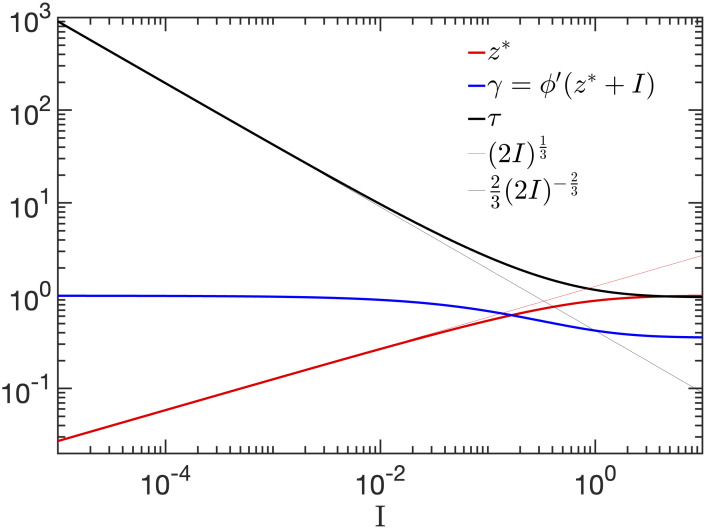
The location of the fixed point (red) is a sensitive (power-law) function of the input; this controls the slope γ of ϕ at the fixed point (blue) and as a result the relaxation time τ (red) also follows a power law. The thin lines are power-law asymptotics to the low I regime: $z^{*}\approx {\left(2I\right)}^{\frac{1}{3}}$ and $\tau \approx \frac{2}{3}{\left(2I\right)}^{-\frac{2}{3}}$. Please see a movie animating this effect in Movie S9. Following ref. 32.

Subtracting the equation for the fixed point, discarding higher orders, and calling $\gamma ≐\phi^{\prime }\left(z^{*}+I\right)$ the slope $\phi^{\prime }$ of the recurrence at the fixed point, we get

$$\delta z_{n+1}=\gamma \delta z_{n}\;\Rightarrow \;\delta z_{n}=\gamma^{n}\delta z_{0}$$

and so the perturbation around the fixed point evolves through powers of γ. Defining the relaxation time τ as the time required for these powers to reach 1/e, we get $\gamma^{\tau }=1/e$ or τ=−1/lnγ. Again, because the fixed point moves very rapidly as a function of I, the value of τ also changes rapidly; it is formally infinite at I=0 because the relaxation at the critical point is algebraic rather than exponential, and follows a power law $\tau \approx I^{-2/3}$. See Fig. 8. As a result, small values of the input have the ability to control the relaxation time over orders of magnitude, in a graded fashion.

### Implementation Notes.

Eq. **1** only needs FFT on a parallelizable platform. For example, in Python using PyTorch, where z is the state, I the input, and Ut the Fourier transform of the kernel U, all three torch.tensor()s of the same shape.


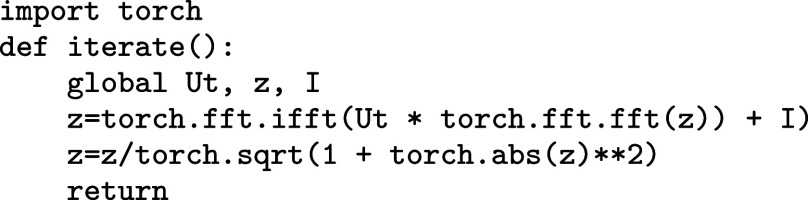


where one would use fft2/ifft2 for two-dimensional tensor layers.

In Matlab it runs


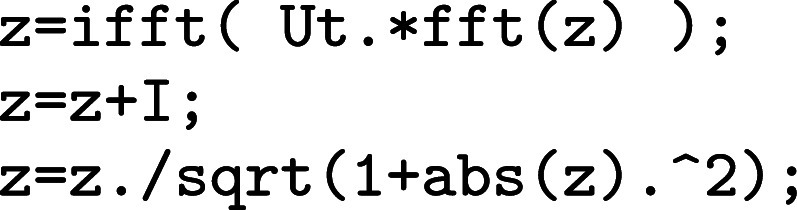


where the arrays can be standard or gpuArray depending on the hardware, and similarly we would use fft2/ifft2 for 2D layers.

### Traveling Waves: Discrete Time vs. Continuous Time.

When shifting the description from continuous time in the form of ordinary differential equations to discrete time in the form of a recurrence or iteration, the natural relationship is one of exponentiation. We review the case of traveling waves, which have recently been implicated in storing short-term memory in RNNs and are similarly central to our construction here (29, 30). Starting with the unidirectional half-wave equation

$$\frac{\partial \psi }{\partial t}=c\frac{\partial \psi }{\partial x}$$

and remembering that the solution of a first-order linear equation $\dot{x}=Mx$ consists of exponentiating, $x\left(t\right)=e^{\mathrm{tM}}x\left(0\right)$, we notice that the derivative operator in the right hand side is linear and hence, the solution should be

$$\psi \left(t,x\right)=e^{ct\frac{\partial }{\partial x}}\psi \left(0,x\right).$$

What is the exponential of a derivative? Expanding the exponential in series we obtain

$$e^{t\frac{\partial }{\partial x}}=1+ct\frac{\partial }{\partial x}+\frac{c^{2}t^{2}}{2}\frac{\partial^{2}}{\partial x^{2}}+\frac{c^{3}t^{3}}{3!}\frac{\partial^{3}}{\partial x^{3}}+\frac{c^{4}t^{4}}{4!}\frac{\partial^{4}}{\partial x^{4}}+\cdots$$

which when applied to ψ(0,x) is nothing more than the Taylor expansion of ψ(0,x+ct), the well-known solution of the equation above. Therefore we say that the exponential of the derivative is a finite translation or, conversely, that the derivative is the infinitesimal generator of translations. In our case, a derivative presents as a real, antisymmetric component of the kernel, and hence its exponential is an orthogonal kernel characterized by eigenvalues with absolute value equal to 1.

## Supplementary Material

Appendix 01 (PDF)

Movie S1. From Figure 2, left panel. No opening between the boxes. The signal cannot cross from top to bottom box.

Movie S2. From Figure 2, right panel. An opening between the boxes permits the signal to cross to the lower box. This illustrates the power to control signal propagation.

Movie S3. From Figure 2 (not shown). Two openings between the boxes. The injected signal now crosses through both openings and since it oscillates with one single frequency, the propagating wave self-interferes in a classic two-slit pattern. The pattern on the lower box thus depends on the signal injected and the geometry of the crossings.

Movie S4. From Figure 3 (not shown). A labyrinthine pattern is created as described to fashion areas where the signal can propagate (black) and areas where it cannot (gray). White noise is injected at a single pixel in the center, waves propagate outwards.

Movie S5. From Figure 3, the evolution shown in the figure. As in S4, but the injected signal is a single-frequency oscillation at an eigenvalue of the kernel (resonant).

Movie S6. From Figure 3 (not shown): To illustrate that the dynamics is independent of the kernel, here we constructed a random anti-Hermitian kernel by generating a 7x7 Gaussian random matrix, and multiplying its symmetric component by *i*. The results are qualitative the same, showing independence from the specifics of the kernel.

Movie S7. From Figure 4. Synchronization ensues within individual connected domains but cannot synchronize across the refractory areas.

Movie S8. From Figure 5, “the Lighthouse”. This simulation depicts directional emission of waves, controlled by the input.

Movie S9. From Figure 8. An animation depicting the motion of the fixed point and the change in slope at the fixed point as a function of the input.

## Data Availability

There are no data underlying this work.
