# Supplementary material for: Input-driven circuit reconfiguration in critical recurrent neural networks
Source: Proc Natl Acad Sci U S A. 2025 Mar 7;122(10):e2418818122. doi: 10.1073/pnas.2418818122 (PMC11912373; doi:10.1073/pnas.2418818122)
Supplement: Supplementary file 1 — Appendix 01 (PDF) [file pnas.2418818122.sapp.pdf]

Supporting Information for

Input-driven circuit reconfiguration in critical recurrent neural networks

Marcelo O. Magnasco[a,1]

Email: mgnsclb@rockefeller.edu

This PDF file includes:

Legends for Movies S1 to S9

Supporting Information Text

Movie S1 (separate file). From Figure 2, left panel. No opening between the boxes. The signal cannot cross from top to bottom box.

Movie S2 (separate file). From Figure 2, right panel. An opening between the boxes permits the signal to cross to the lower box. This illustrates the power to control signal propagation.

Movie S3 (separate file). From Figure 2 (not shown). Two openings between the boxes. The injected signal now crosses through both openings and since it oscillates with one single frequency, the propagating wave self-interferes in a classic two-slit pattern. The pattern on the lower box thus depends on the signal injected and the geometry of the crossings.

Movie S4 (separate file). From Figure 3 (not shown). A labyrinthine pattern is created as described to fashion areas where the signal can propagate (black) and areas where it cannot (gray). White noise is injected at a single pixel in the center, waves propagate outwards.

Movie S5 (separate file). From Figure 3, the evolution shown in the figure. As in S4, but the injected signal is a single-frequency oscillation at an eigenvalue of the kernel (resonant).

Movie S6 (separate file). From Figure 3 (not shown): To illustrate that the dynamics is independent of the kernel, here we constructed a random anti-Hermitian kernel by generating a 7x7 Gaussian random matrix, and multiplying its symmetric component by *i*. The results are qualitative the same, showing independence from the specifics of the kernel.

Movie S7 (separate file). From Figure 4. Synchronization ensues within individual connected domains but cannot synchronize across the refractory areas.

Movie S8 (separate file). From Figure 5, “the Lighthouse”. This simulation depicts directional emission of waves, controlled by the input.

Movie S9 (separate file). From Figure 8. An animation depicting the motion of the fixed point and the change in slope at the fixed point as a function of the input.
